# Spousal Similarities in Cardiovascular Risk Factors in Northern China: A Community-Based Cross-Sectional Study

**DOI:** 10.3389/ijph.2023.1605620

**Published:** 2023-02-21

**Authors:** Binbin Lin, Li Pan, Huijing He, Yaoda Hu, Ji Tu, Ling Zhang, Ze Cui, Xiaolan Ren, Xianghua Wang, Jing Nai, Guangliang Shan

**Affiliations:** ^1^ Department of Epidemiology and Statistics, Institute of Basic Medical Sciences Chinese Academy of Medical Sciences, School of Basic Medicine Peking Union Medical College, Beijing, China; ^2^ Department of Epidemiology and Health Statistics, School of Public Health, Capital Medical University, Beijing, China; ^3^ Department of Chronic and Non-Communicable Disease Prevention and Control, Hebei Provincial Center for Disease Control and Prevention, Shijiazhuang, China; ^4^ Department of Chronic and Non-Communicable Disease Prevention and Control, Gansu Provincial Center for Disease Control and Prevention, Lanzhou, China; ^5^ Institute of Biomedical Engineering, Chinese Academy of Medical Sciences, Tianjin, China; ^6^ Clinical Laboratory, Beijing Hepingli Hospital, Beijing, China

**Keywords:** cardiovascular risk factors, lifestyles, cardiometabolic diseases, spousal concordance, metabolic indicators

## Abstract

**Objectives:** The aim of this study was to explore spousal similarities in cardiovascular risk factors in northern China.

**Methods:** We conducted a cross-sectional study of married couples from Beijing, Hebei, Gansu, and Qinghai provinces between 2015 and 2019. A total of 2,020 couples were included in the final analyses. The spousal similarities for metabolic indicators and cardiovascular risk factors (including lifestyle factors and cardiometabolic diseases) were evaluated using Spearman’s correlation and logistic regression analyses, respectively.

**Results:** All metabolic indicators showed positive spousal correlations (*p* < 0.001), with the strongest for fasting blood glucose (*r* = 0.30) and the lowest for high-density lipoprotein cholesterol (*r* = 0.08). Significant husband-wife associations were observed for several cardiovascular risk factors except for hypertension in multivariable models, with the strongest association for physical inactivity (odds ratios with 95% confidence intervals of 3.59 [2.85, 4.52] and 3.54 [2.82, 4.46] for husbands and wives, respectively). In addition, the interaction of age with spousal overweight/obesity status was statistically significant, and the association was stronger in people ≥50 years.

**Conclusion:** There were spousal similarities in cardiovascular risk factors. The finding may have public health implications that targeted screening and interventions for spouses of people with cardiovascular risk factors.

## Introduction

The occurrence of cardiovascular diseases continues to increase in China due to the dual pressures of population ageing and the steady rise in the prevalence of metabolic risk factors. In 2019, the number of people with cardiovascular diseases was nearly 330 million, with two out of five deaths attributable to cardiovascular diseases [1]. Both genetic and environmental factors may contribute to the development of cardiovascular diseases [2]. The well-recognized environmental risk factors for cardiovascular diseases are smoking, alcohol consumption, high body mass index (BMI), high blood pressure, high cholesterol, and high fasting blood glucose [3]. In addition, the prevalence of hyperuricemia in the Chinese population has significantly increased in recent decades [4]. Several studies have suggested that hyperuricemia significantly increases the risk of cardiovascular diseases through the potential mechanism of inducing inflammation, oxidative stress, and subsequent endothelial dysfunction [5, 6]. Most cardiovascular risk factors are modifiable or manageable, and effective interventions for individuals with these risk factors may reduce the burden of cardiovascular diseases [7].

Couples are not genetically related but are likely to face the same health problems due to sharing environmental factors, adopting similar behaviors, or experiencing assortative mating (non-random partner selection based on the similarity of observable characteristics) [8]. There have been several studies on the spousal aggregation of obesity [9], hypertension [10, 11], diabetes [12, 13], depression [14], and cancer [15]. Likewise, the behaviors between spouses might influence each other, which suggests that interventions targeted at couples may be more effective than those targeted at individuals [9]. A few studies have demonstrated concordance of cardiovascular risk factors between Chinese couples [2, 16, 17]. However, two of these studies were with participants from cities in southern China [2, 17]. Cultural differences between northern and southern China may affect the concordance of couples’ lifestyles. Liao et al. focused only on middle-aged and elderly couples and merely studied the concordance of chronic disease, not lifestyles [16]. Moreover, to the best of our knowledge, concordance of hyperuricemia between couples has not been reported.

The aim of our study is to investigate spousal associations for modifiable lifestyles (smoking, alcohol drinking, leisure-time physical activity, and overweight/obesity) and cardiometabolic diseases (hypertension, diabetes mellitus, dyslipidemia, and hyperuricemia), which are major risk factors for cardiovascular disease, in married couples from four provinces in northern China. We also assessed whether the concordance differed by age.

## Methods

### Study Population

The current study was based on the China National Health Survey (CNHS) and the cohort study of the general population in the Beijing-Tianjin-Hebei (an abbreviation of the Chinese name is Jing-Jin-Ji) area (J-J-J Cohort Study). The CNHS is a nationwide cross-sectional study conducted from 2012 to 2017, and the J-J-J Cohort Study is a population-based prospective cohort study initiated in 2017. Details of the CNHS and the J-J-J Cohort Study designs and methods have been described elsewhere [18, 19]. In brief, the CNHS and the J-J-J Cohort Study investigated community populations aged 18 years and above, collecting data on demographic and socioeconomic information, lifestyle factors, anthropometric measures, laboratory tests, and clinical profiles to explore risk factors for major chronic diseases. The CNHS and the J-J-J Cohort Study were approved by the ethics committee of the Institute of Basic Medical Sciences, Chinese Academy of Medical Sciences. All participants provided written informed consent.

In this study, we used data from Qinghai, Gansu, and Hebei provinces, three CNHS survey sites surveyed from 2015 to 2017, and Beijing, one of the J-J-J Cohort Study survey sites conducted in 2019, as additional information on family relationships was collected at these four survey sites. We identified 2,073 couples who were married at the time of the interview and further excluded couples if either member had missing data for sociodemographic information (age, sex, education, and income), lifestyle factors (smoking status, alcohol consumption, and exercise), medical history (hypertension and diabetes), BMI, blood pressure, and blood biochemistry. A total of 2,020 couples were included in the current analyses. Supplementary Figure S1 shows a flow chart for selecting participants.

### Data Collection

The questionnaire and equipment were uniform and the criteria was consistent for the measurements that were used by the CNHS and the J-J-J Cohort Study. All investigators underwent a training program to guarantee their capability to conduct precise data collection. Information on sociodemographic characteristics, lifestyle factors, and personal and family medical history was obtained from a standard questionnaire through face-to-face interviews. To ensure data accuracy, questionnaires were checked and verified by inspectors after the completion of each questionnaire. All participants were asked, “Has your spouse also come to participate in the survey?” Furthermore, the ID and name of the participant’s spouse were collected if they replied “yes” to the first question. Couples could be combined using the family ID variable.

A physical examination, including anthropometry measurements (height and body weight) and blood pressure measurements, was conducted by well-trained staff. Body weight and height were measured using standard procedures with the participants wearing light clothes and no shoes. BMI was calculated as body weight (kg) divided by height (m) squared. Blood pressure was measured 3 times using an automated electronic device (OMRON, HEM-907). The mean value of the 3 readings was used for analysis. Fasting blood samples were collected from participants for measurements of fasting blood glucose (FBG), total cholesterol (TC), high-density lipoprotein cholesterol (HDL-C), low-density lipoprotein cholesterol (LDL-C), triglycerides (TG), and serum uric acid (UA) *via* a standard protocol.

### Definition of Cardiovascular Risk Factors

#### Modifiable Lifestyle Factors

Lifestyle behaviors, including smoking, alcohol consumption, and physical activity, were defined according to the validated self-report questionnaires. Subjects were classified as current smokers or non-smokers. Drinking status was categorized into current drinkers or non-drinkers. Physical activity level was divided into two categories according to the frequency of leisure time exercise in the last year. Physical inactivity was defined as exercising less than once per week. Overweight/obesity was defined as BMI ≥24 kg/m^2^ based on criteria for Chinese individuals [20].

#### Cardiometabolic Diseases

Participants who reported having been diagnosed with hypertension by a doctor or who had an average measured systolic blood pressure (SBP) ≥ 140 mmHg or diastolic blood pressure (DBP) ≥ 90 mmHg were defined as having hypertension. Diabetes was defined as having been diagnosed with diabetes by a doctor or having a measured FBG concentration ≥7.0 mmol/L. According to Chinese guidelines on the prevention and treatment of dyslipidemia in adults, dyslipidemia was defined as having at least one of the following: high TC (≥6.2 mmol/L), low HDL-C (<1.0 mmol/L), high LDL-C (≥4.1 mmol/L), and high TG (≥2.3 mmol/L) [21]. Hyperuricemia was defined as serum UA > 360 μmol/L in women and serum UA > 420 μmol/L in men.

### Exposure and Outcome Variables and Covariates

The exposure variables in our study were spousal cardiovascular risk factors, including current smoking, current drinking, physical inactivity, overweight/obesity, hypertension, diabetes, dyslipidemia, and hyperuricemia. The corresponding cardiovascular risk factors for individuals were defined as the outcome. Sociodemographic covariates included age (20–29, 30–39, 40–49, 50–59, 60–69, ≥70 years), education level (middle school or below, high school or above), annual income (<24,000 RMB, ≥24,000 RMB), and geographic region (Qinghai, Gansu, Hebei, and Beijing). The abovementioned lifestyle factors and a family history of hypertension or diabetes were also included as covariates in the models for cardiometabolic diseases. A family history of hypertension or diabetes was defined as having at least one first-degree relative with hypertension or diabetes.

### Statistical Analysis

Characteristics of the study population were presented as the mean ± SD or median (IQR) for continuous variables and the number (frequency) for categorical variables according to sex. Student’s t tests or Wilcoxon rank sum tests were used to compare the differences between groups for continuous variables. The *χ* ^*2*^ test was used to compare the differences between groups for categorical variables. Concordance for cardiovascular risk factors was defined as a case in which both spouses had the same response for a category of variables.

The univariate Spearman correlation coefficient (*r*) and the Phi coefficient were used to assess the correlations of continuous and categorical variables within couples, respectively. Spousal correlations for metabolic indicators (BMI, SBP, DBP, FBG, TC, HDL-C, LDL-C, TG, and UA) might emerge because of the associations of these indicators with age. Therefore, we fitted a regression of metabolic indicators against age and derived the residual for these indicators adjusted for individuals’ age. We then used the residuals to calculate the spousal correlations in the metabolic indicators; namely, the correlation coefficients were corrected for the age of both partners (Model 1). Since previous studies reported that BMI is a surrogate for assortative mating [22], we further adjusted the BMI of both spouses in Model 2 using the same method.

The *χ* ^*2*^ test was performed to explore the crude association of cardiovascular risk factors between couples. The odds ratios (ORs) and their corresponding 95% confidence intervals (CIs) were estimated by age-adjusted and multivariable-adjusted logistic regression models to indicate the spousal association for each given cardiovascular risk factor. The multivariable model of lifestyle factors adjusted the age, education, annual income, and geographic regions of the individuals. The multivariable model of cardiometabolic disease was further adjusted for individuals’ smoking, drinking, leisure-time physical activity, and overweight/obesity status. Family history of hypertension and diabetes was additionally adjusted in the models of hypertension and diabetes, respectively. Spousal similarity was defined as *r* > 0 and its corresponding *p* value <0.05 for continuous variables and ORs >1 and their corresponding 95% CIs excluding 1 for categorical variables.

To explore potential changes in spousal similarities with age, roughly representing marriage duration, subgroup analyses were performed according to the individuals’ age (20–50 years, ≥50 years). Multiplicative interaction was calculated by cross-product interaction terms in multivariable logistic regression models. A sensitivity analysis was performed, excluding couples with an age difference ≥5 years, to evaluate the robustness of the results.

All analyses were performed separately for husbands and wives. The significance level was set as a 2-sided *p* value < 0.05. SAS 9.4 (SAS Institute Inc., Cary, NC, United States.) was used to conduct all analyses.

## Results

The characteristics of the women and men are presented in Table 1. A total of 2,020 married couples, with a mean (SD) age of 55.4 (12.1) years among men and 53.7 (11.9) years among women, were included in the study. The study population was geographically diverse (32.48% from Beijing, 28.32% from Hebei, 31.68% from Gansu, and 7.52% from Qinghai). Husbands were more likely than their wives to have higher levels of education, annual income, BMI, SBP, DBP, FBG, and UA.

**TABLE 1:** Basic characteristics of the study population by Sex. Qinghai, Gansu, Hebei, and Beijing, China, 2015–2019.

Variables	Overall (N = 4,040)	Male (*n* = 2,020)	Female (*n* = 2,020)	*p* value
Demographic characteristics				
Age (years), mean (SD)	54.5 ± 12.0	55.4 ± 12.1	53.7 ± 11.9	<0.0001
Geographic regions, n (%)				—
Beijing	1,312 (32.48)	656 (32.48)	656 (32.48)	
Hebei	1,144 (28.32)	572 (28.32)	572 (28.32)	
Gansu	1,280 (31.68)	640 (31.68)	640 (31.68)	
Qinghai	304 (7.52)	152 (7.52)	152 (7.52)	
Annual income (RMB), median (IQR)	24,000 (12,000–42,000)	30,000 (13,333–48,000)	24,000 (12,000–36,000)	<0.0001
Educated to high school or above, n (%)	1,400 (34.7)	762 (37.7)	638 (31.6)	<0.0001
Metabolic indicators				
BMI (kg/m^2^), mean (SD)	25.0 ± 3.5	25.3 ± 3.4	24.8 ± 3.5	<0.0001
SBP (mmHg), mean (SD)	128.0 ± 18.2	130.2 ± 16.8	125.8 ± 19.2	<0.0001
DBP (mmHg), mean (SD)	76.3 ± 10.9	78.5 ± 10.7	74.1 ± 10.6	<0.0001
FBG (mmol/L), mean (SD)	5.9 ± 1.7	6.1 ± 1.9	5.8 ± 1.5	<0.0001
TC (mmol/L), mean (SD)	4.8 ± 1.0	4.8 ± 1.0	4.9 ± 1.1	<0.0001
HDL-C (mmol/L), mean (SD)	1.3 ± 0.3	1.2 ± 0.3	1.3 ± 0.3	<0.0001
LDL-C (mmol/L), mean (SD)	2.8 ± 0.8	2.8 ± 0.8	2.8 ± 0.8	0.0010
TG (mmol/L), median (IQR)	1.5 (1.1–2.2)	1.5 (1.1–2.3)	1.5 (1.1–2.1)	<0.0001
UA (μmol/L), mean (SD)	322.4 ± 88.2	362.7 ± 87.7	282.1 ± 67.9	<0.0001

Abbreviations: BMI, body mass index; DBP, diastolic blood pressure; SBP, systolic blood pressure; FBG, fasting blood glucose; TC, total cholesterol; HDL-C, high-density lipoprotein cholesterol; LDL-C, low-density lipoprotein cholesterol; TG, triglycerides; UA, uric acid; SD, standard deviation; IQR, interquartile range.

**FIGURE 1:**
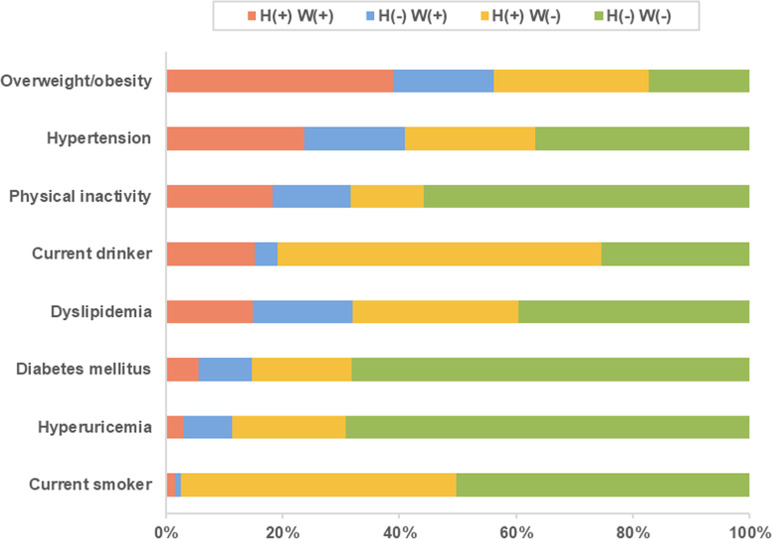
Percentage of couples in concordance categories of cardiovascular risk factors. H+ W+ indicates couples in which both the husband and wife have the characteristic; H- W-**,** couples in which neither have it; H- W+ and H+ W- indicate discordant pairs. Qinghai, Gansu, Hebei, and Beijing, China, 2015–2019.

The within-couple concordance (the proportion of couples in which both or neither of the spouses had a certain risk factor) for the cardiovascular risk factors ranged from a low of 40.70% for current drinkers to a high of 74.1% for leisure-time physical activity (Figure 1). The proportion of couples who were both overweight/obese and hypertensive accounted for approximately 39% and 24% of couples, respectively. The correlation of leisure-time physical activity within couples was the strongest among cardiovascular risk factors (phi = 0.3972, see Supplementary Table S1).

Table 2 shows that there were significant correlations between spouses in BMI (*r* = 0.17; *p* < 0.001), SBP (*r* = 0.26; *p* < 0.001), DBP (*r* = 0.11; *p* < 0.001), FBG (*r* = 0.30; *p* < 0.001), TC (*r* = 0.16; *p* < 0.001), HDL-C (*r* = 0.08, *p* < 0.001), LDL-C (*r* = 0.16, *p* < 0.001), TG (*r* = 0.13, *p* < 0.001), and UA (*r* = 0.14; *p* < 0.001). After adjusting for the age and BMI of both spouses, the correlations remained significant for all traits, but the spousal correlations for SBP and FBG decreased substantially (SBP: from 0.26 to 0.12; FBG: from 0.30 to 0.17), whereas the spousal correlations for TG and UA increased (TG: from 0.13 to 0.19; UA: from 0.14 to 0.18). As shown in Supplementary Table S2, the correlations of BMI, SBP, DBP, and LDL-C between couples were stronger in those aged 50 years and older, but the correlation of HDL-C and UA was stronger in young couples (20–50 years).

**TABLE 2:** Spearman correlations of metabolic indicators within couples. Qinghai, Gansu, Hebei, and Beijing, China, 2015–2019.

Metabolic indicators	Unadjusted		Model 1		Model 2	
	*r*	*p* Value	*r*	*p* Value	*r*	*p* Value
BMI	0.17	<0.001	0.17	<0.001	—	—
SBP	0.26	<0.001	0.16	<0.001	0.12	<0.001
DBP	0.11	<0.001	0.12	<0.001	0.11	<0.001
FBG	0.30	<0.001	0.21	<0.001	0.17	<0.001
TC	0.16	<0.001	0.16	<0.001	0.15	<0.001
HDL-C	0.08	<0.001	0.08	<0.001	0.09	<0.001
LDL-C	0.16	<0.001	0.15	<0.001	0.15	<0.001
TG	0.13	<0.001	0.16	<0.001	0.19	<0.001
UA	0.14	<0.001	0.18	<0.001	0.18	<0.001

Abbreviations: BMI, body mass index; SBP, systolic blood pressure; DBP, diastolic blood pressure; FBG, fasting blood glucose; TC, total cholesterol; HDL-C, high-density lipoprotein cholesterol; LDL-C, low-density lipoprotein cholesterol; TG, triglycerides; UA, uric acid. Model 1: Adjusted for the age of both partners. Model 2: Adjusted for age and BMI of both partners.

Significant spousal associations were observed in four lifestyle factors after adjusting for individuals’ age, education, annual income, and geographic regions (Table 3). Men whose wives were current smokers or current drinkers had significantly higher odds of being current smokers (OR: 1.90; 95% CI: 1.07, 3.38) or current drinkers (OR:1.59; 95% CI: 1.20, 2.10), and *vice versa*. Physical inactivity showed the strongest spousal association among cardiovascular risk factors, with ORs (95% CIs) of 3.59 (2.85, 4.52) and 3.54 (2.82, 4.46) for husbands and wives, respectively. Participants with spouses who were overweight/obese were at an increased risk of being overweight/obese (husbands: OR = 1.37 [95% CI: 1.13, 1.66]; wives: OR = 1.40 [95% CI: 1.15, 1.70]).

**TABLE 3:** Spousal associations for cardiovascular risk factors by logistic regression analysis. Qinghai, Gansu, Hebei, and Beijing, China, 2015–2019.

Spouses’ status	Husbands		Wives	
	Model 1	Model 2	Model 1	Model 2
Modifiable lifestyles^a^				
Current smoker				
No (ref)	1	1	1	1
Yes	1.75 (0.99, 3.08)	1.90 (1.07, 3.38)	1.73 (0.98, 3.06)	1.81 (1.02, 3.23)
Current drinker				
No (ref)	1	1	1	1
Yes	1.62 (1.23, 2.14)	1.59 (1.20, 2.10)	1.61 (1.22, 2.12)	1.56 (1.18, 2.07)
Physical inactivity				
No (ref)	1	1	1	1
Yes	4.96 (4.00, 6.15)	3.59 (2.85, 4.52)	4.93 (3.97, 6.12)	3.54 (2.82, 4.46)
Overweight/obesity				
No (ref)	1	1	1	1
Yes	1.53 (1.27, 1.85)	1.37 (1.13, 1.66)	1.50 (1.24, 1.82)	1.40 (1.15, 1.70)
Cardiometabolic disease^b^				
Hypertension				
No (ref)	1	1	1	1
Yes	1.42 (1.16, 1.74)	1.21 (0.97, 1.51)	1.34 (1.09, 1.64)	1.09 (0.87, 1.37)
Diabetes				
No (ref)	1	1	1	1
Yes	1.82 (1.39, 2.38)	1.73 (1.29, 2.31)	1.82 (1.38, 2.39)	1.55 (1.16, 2.07)
Dyslipidemia				
No (ref)	1	1	1	1
Yes	1.32 (1.09, 1.60)	1.29 (1.06, 1.57)	1.34 (1.10, 1.62)	1.28 (1.05, 1.57)
Hyperuricemia				
No (ref)	1	1	1	1
Yes	1.47 (1.07, 2.02)	1.41 (1.01, 1.96)	1.49 (1.09, 2.04)	1.42 (1.02, 1.97)

Abbreviations: ref, References. Values are presented as odds ratio (95% confidence interval) for having identical risk factors among spouses. Model 1 adjusted for age.

^a^ Model 2 further adjusted for education, annual income, and geographic regions.

^b^ Model 2 further adjusted for education, annual income, geographic regions, smoking, drinking, leisure-time physical activity, and overweight/obesity. Family history of hypertension and diabetes was additionally adjusted in the models of hypertension and diabetes, respectively.

There were statistically significant associations between the wives’ health conditions and the corresponding health conditions in their husbands, with multivariable-adjusted ORs (95% CIs) of 1.73 (1.29, 2.31) for diabetes, 1.29 (1.06, 1.57) for dyslipidemia, and 1.41 (1.01, 1.96) for hyperuricemia. Husbands’ health condition statuses had similar effects on their wives. However, the wife-husband association was not significant for hypertension in the multivariable model. In addition, the spousal associations varied in different types of dyslipidemia in the multivariable model, with significant wife-husband associations observed in low HDL-C and high TG (Supplementary Table S3). The women whose spouses had high LDL-C were at an increased risk of having high LDL-C than those whose spouses did not have high LDL-C, while the spousal association was not significant in men.

**FIGURE 2:**
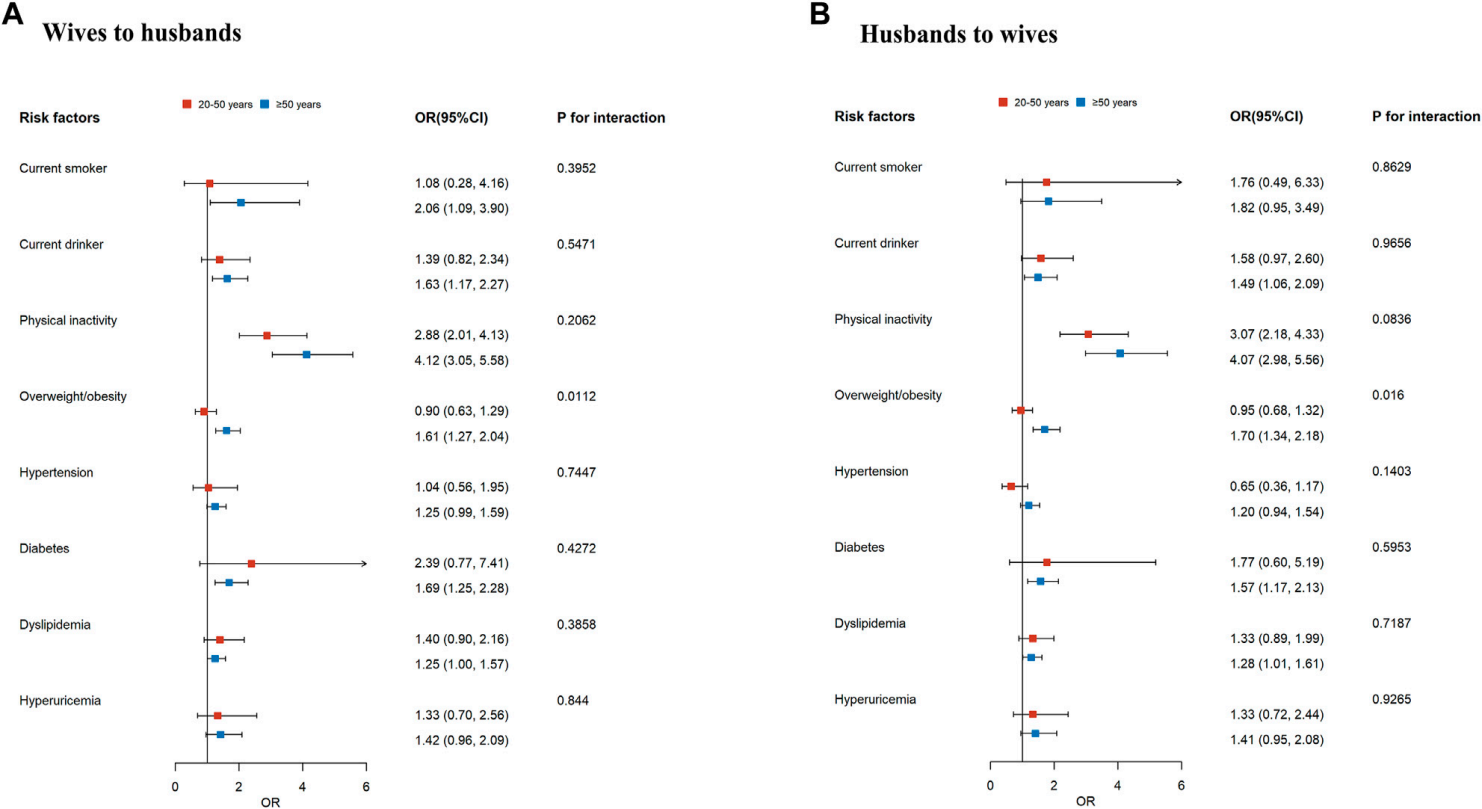
The forest plot of spousal associations for cardiovascular risk factors by age. The plot shows the adjusted odds ratios (ORs) and 95% confidence intervals (CIs) of the association between each given cardiovascular risk factor for individuals with the same condition as spouses. The reference to all risk factors is “no.” Wives’ influence on husbands **(A)**. Husbands’ influence on wives **(B)**. Models for current smoker, current drinker, physical inactivity, and overweight/obesity were adjusted for individuals’ age, education, annual income, and geographic regions. Models for hypertension, diabetes, dyslipidemia, and hyperuricemia were further adjusted for individuals’ smoking status, drinking status, physical activity, and overweight/obesity. Family history of hypertension and diabetes was additionally adjusted in the models of hypertension and diabetes, respectively. Qinghai, Gansu, Hebei, and Beijing, China, 2015–2019.

Figure 2 shows that the husband-wife associations for cardiovascular risk factors did not differ appreciably according to age group, except for overweight/obesity. We observed a significant interaction between age and overweight/obesity status (for men, *P* for interaction = 0.0112; for women, *P* for interaction = 0.016). The association was stronger in individuals aged ≥50 years old than in those aged <50 years old (OR: 1.61 [95% CI: 1.27, 2.04] compared with 0.90 [0.63, 1.29] for men; 1.70 [1.34, 2.18] compared with 0.95 [0.68, 1.32] for women), which suggests that having a spouse with overweight/obesity in middle-aged and elderly individuals would have a higher risk of becoming overweight/obese. The spousal associations for physical inactivity were significant across age groups. To test the robustness of our findings, we excluded couples with an age difference of ≥5 years and found that the results were not substantially changed (Supplementary Table S4).

## Discussion

In this community-based, cross-sectional study, we found statistically significant spousal correlations in metabolic indicators among Chinese couples. Significant wife-husband associations were observed for several cardiovascular risk factors, with stronger associations for behavioral risk factors. No statistically significant association was found for hypertension after adjusting for their personal risk factors. There were no sex differences in our findings.

Consistent with previous studies [2, 17], husbands had a higher level of education but also had higher levels of BMI, SBP, DBP, FBG, and UA than their wives, which may be attributed to the traditional gender norm that women tend to marry men with a higher level of education [23] and sex differences in the distribution of these metabolic indicators [24]. Additionally, the mean age of husbands was slightly older than that of wives, and BMI, SBP, DBP, and FBG significantly increased with age [25]. We found statistically significant but generally weak and modest spousal correlations for metabolic indicators. The magnitude of these correlations was in line with previous studies [26, 27]. After adjusting for both partners’ age and BMI, the correlation coefficients for SBP and FBG decreased significantly, indicating that spousal correlations for the two traits were partly due to the within-couple correlations for age and BMI (age: *r* = 0.97; BMI: *r* = 0.17 in our study). Nakaya et al. found spousal similarities in cardiometabolic risk factors among 5,391 random male-female pairs with similar ages, but the significant correlations disappeared after adjustment for age [28]. However, the correlation coefficients between couples for metabolic indicators remained significant after adjusting for both partners’ age and BMI in our study, suggesting that other important environmental factors may contribute to spousal similarities in these indicators. Wilson et al. found that health behavior concordance (e.g., diet and sleep) and marital quality affect cardiometabolic similarity between couples [29]. We admit that some small effect sizes (e.g., the spousal correlation of 0.08 for HDL-C) may not be very meaningful clinically, but we suggest that shared environmental factors may play an important role in spousal similarities for metabolic profiles.

Our results demonstrated a positive association for lifestyles among couples, consistent with previous studies [17, 30]. We found that the risk of being a current smoker when having a currently smoking spouse was more than 1.8 times that of having a current non-smoking spouse. However, Dutch and American couples showed substantially stronger associations for current smokers, with ORs (95% CIs) of 6.9 (6.3, 7.5) and 5.4 (4.7, 6.3), respectively [26, 31]. Notably, 48.7% and 2.6% of Chinese husbands and wives were current smokers, respectively, compared with 15.5% and 11.4% in Dutch couples [26] and 12.6% and 6.5% in American couples [31]. Similarly, Dutch couples with smaller sex differences in the prevalence of current drinking had a stronger association for current drinking (OR: 5.14; 95 CI%: 4.70, 5.61) than our study (1.59; 1.20, 2.10). Therefore, the degree of spousal concordance for current smoking and drinking may be strongly influenced by the disparities in current smoking and drinking prevalence across sex. Further, the phenomenon may be attributed to cultural differences in the acceptance and freedom of smoking and alcohol consumption among women and men [32].

Increased intake of energy-dense foods and decreased physical activity appear to account for the dramatically increased prevalence of obesity throughout the past few decades [33]. In the current study, the highest proportion of cardiovascular risk factors shared by both spouses was overweight/obesity, which accounted for approximately 40%. The odds of being overweight/obese were nearly 1.4 times higher among individuals whose spouses were overweight/obese than among those whose spouses were not overweight/obese, which was in line with earlier evidence [14]. The spousal concordance for overweight/obesity may be attributed to couples’ similar diet and physical activity habits [9]. Middle-aged and elderly couples with a stronger spousal association for overweight/obesity may become the key group for weight management.

A meta-analysis suggested that people are likely to have hypertension (OR: 1.41; 95% CI: 1.08, 1.45) if their spouse has hypertension [34]. However, most of the studies included in the meta-analysis adjusted only for demographic characteristics variables such as age and education. We found that the age-adjusted husband-wife association for hypertension was statistically significant, but the association was not statistically significant after further adjusting for individuals’ education level, income, geographic locations, and lifestyle factors, which may imply that the spousal association for hypertension can be mainly explained by spousal concordance for these covariates. Spouses of those affected by diabetes are at a more than 1.5-fold higher risk of diabetes after adjusting for individuals’ risk factors, which was in line with previous studies [10, 27].

Uric acid is the end product of purine metabolism in the human body. Although the causal relationship between uric acid and cardiovascular disease remains unclear, many epidemiological studies have suggested that hyperuricemia is strongly associated with cardiovascular disease [5, 35]. To our knowledge, this is the first study to report spousal similarity in hyperuricemia. The odds of having hyperuricemia when the spouse had hyperuricemia were significantly increased in our study. We also found that the correlation coefficient of UA within young couples was greater than that of older couples. Notably, a recent study reported that the rising prevalence of hyperuricemia in the younger population [36], which implies that we should improve the management of uric acid levels in young couples.

The following two theories have been widely publicized in an attempt to explain the spousal concordance of cardiovascular risk factors: (1) the observed associations may be due to the tendency to select spouses based on a preference for similar characteristics (assortative mating) or (2) the impact of the shared environments and convergence in lifestyles during long-term cohabitation [2, 34]. In our opinion, having a spouse with a cardiometabolic disease increases the risk of developing the same disease due to an indirect association. First, couples had similar sociodemographic characteristics, such as similar age, which is one of the most significant risk factors for cardiometabolic diseases. Second, couples are likely to adopt similar lifestyles, which are well-recognized environmental risk factors for cardiometabolic diseases. Helga et al. reported that spousal concordance in lifestyles was due to assortative mating and convergence over time [37]. For example, individuals more often choose a spouse with similar exercise habits. Over the course of long-term cohabitation, spousal interactions and shared environmental resources (e.g., access to community sports facilities) may also lead to similar spousal physical activity participation [26]. Finally, the husband-wife associations remained significant after adjusting for sociodemographic characteristics and lifestyles, which suggests that important unobserved factors shared by couples impact their likelihood of developing the same diseases, such as dietary patterns. The two theories jointly explain the spousal resemblance in cardiovascular risk factors, and it is difficult to determine the most important factors.

Our findings have important public health implications. Spousal similarities in cardiovascular risk factors were observed among couples from northern China, and there was a higher burden of cardiovascular disease [38]. Smoking, alcohol consumption, physical inactivity, and overweight/obesity are well-known modifiable risk factors for cardiovascular diseases [3]. A previous study suggested that individuals are likely to make similar changes when their spouses improve their behaviors (i.e., quit smoking, quit drinking, start to exercise) [30]. It has been shown that programs targeted at couples can be more effective in improving health behavior than individual interventions [39], which may provide new ideas for cardiovascular disease prevention strategies in China. For example, couple-based lifestyle interventions can be implemented in the community to encourage couples to participate in regular physical activity and weight control together. Additionally, regular screening may be recommended for the spouses of patients with cardiometabolic diseases and interventions as early as possible to reduce the risk of these diseases in their spouses. Exploring the pattern and effects of couple-based intervention programs may be increasingly important in the future.

### Limitations

There were several limitations in this study. The marriage duration of couples was unavailable, so we could not determine the long-term and short-term effects of marriage on cardiovascular risk factors. However, a previous study reported that age and relationship length are closely correlated (*r* = 0.78) [29]. Therefore, a stratified analysis of age was performed to roughly assess the effect of marital length on the associations of cardiovascular risk factors between couples in our study. Second, since the study population was from four northern provinces of China, the results could not represent the situation in other regions of China. Nevertheless, it could suggest that we should pay attention to the health status of individuals whose spouses have cardiovascular risk factors. Finally, a cross-sectional design cannot assess the associations of cardiovascular risk factors within couples over time.

### Conclusion

The aggregation of risk factors for cardiovascular disease in couples from northern China was depicted in this study. The results suggested that if wives had unhealthy lifestyles and cardiometabolic diseases, then their husbands were susceptible to the same behavior or disease and *vice versa*. Overall, these observations may imply that intervention and regular screening of spouses of patients with cardiovascular risk factors are warranted.
