# Supplementary material for: Spousal Similarities in Cardiovascular Risk Factors in Northern China: A Community-Based Cross-Sectional Study
Source: Int J Public Health. 2023 Feb 21;68:1605620. doi: 10.3389/ijph.2023.1605620 (PMC9988901; doi:10.3389/ijph.2023.1605620)
Supplement: Supplementary file 1 [file DataSheet1.docx]

**ORIGINAL ARTICLE**

*International Journal of Public Health*

**Spousal Similarities in Cardiovascular Risk Factors in Northern China:** **A Community-based Cross-sectional Study**

**Supplemental Material**

**Figure S1 |** Flowchart of selecting participants**.** Qinghai, Gansu, Hebei, and Beijing, China, 2015-2019

**Table S1 |** Distribution of cardiovascular risk factors in couples. Qinghai, Gansu, Hebei, and Beijing, China, 2015-2019

**Table S2 |** Raw spearman correlations of cardiovascular risk factors within couples by age. Qinghai, Gansu, Hebei, and Beijing, China, 2015-2019

**Table S3 |** Spousal associations for different types of dyslipidemia by logistic regression analysis. Qinghai, Gansu, Hebei, and Beijing, China, 2015-2019

**Table S4 |** Sensitivity analysis on spousal associations for cardiovascular risk factors. Qinghai, Gansu, Hebei, and Beijing, China, 2015-2019

**
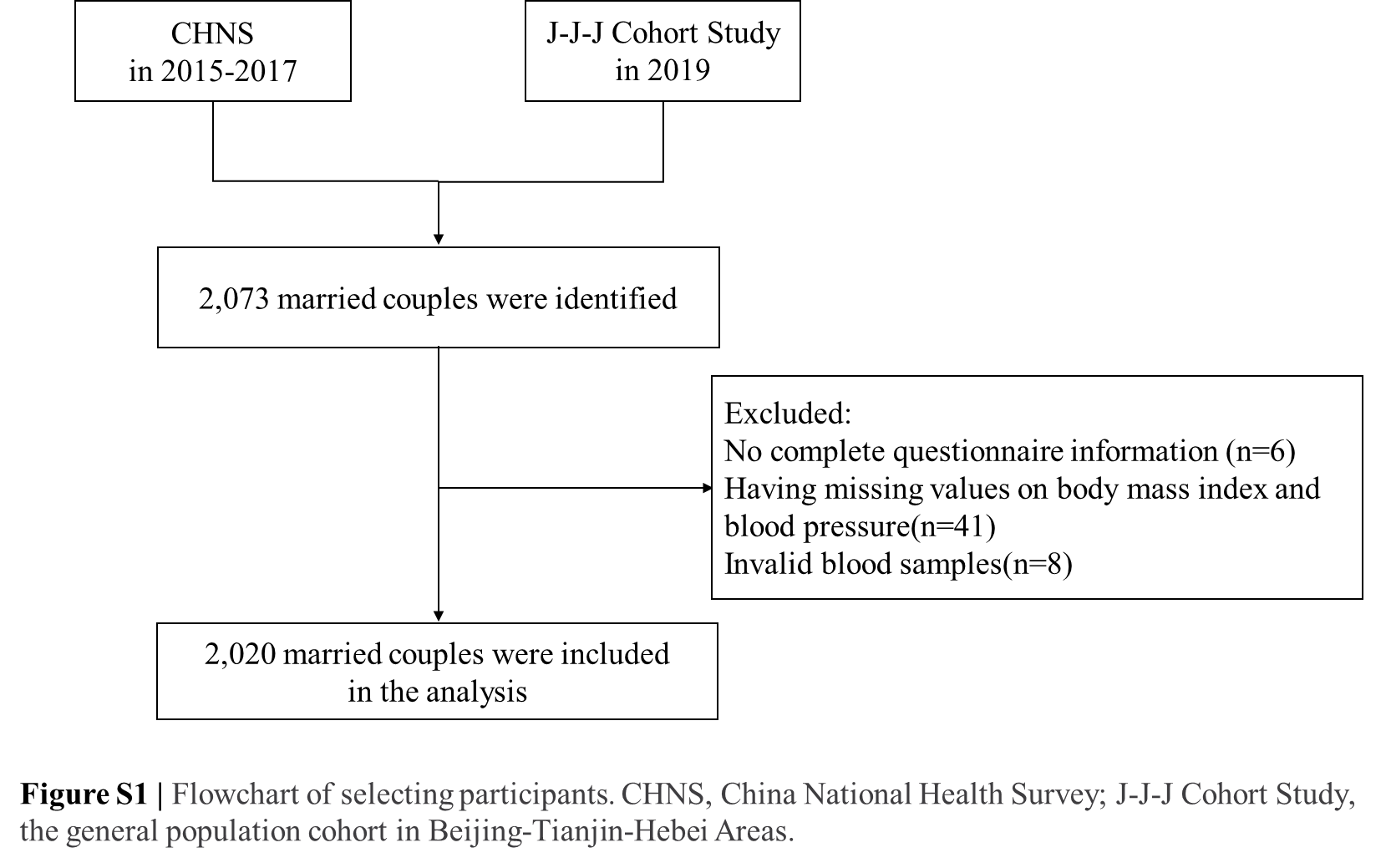
**

**Figure S1 |** Flowchart of selecting participants. CHNS, China National Health Survey; J-J-J Cohort Study, the cohort study of the general population in the Beijing-Tianjin-Hebei area. Qinghai, Gansu, Hebei, and Beijing, China, 2015-2019

**Table S1 |** Distribution of cardiovascular risk factors in couples.

Qinghai, Gansu, Hebei, and Beijing, China, 2015-2019

| **Factors** | **Husband (n=2,020)** | **Wives (n=2,020)** | **Phi** | **Crude OR (95% CI)** | ***P* value** |
| --- | --- | --- | --- | --- | --- |
| Modifiable lifestyles |  |  |  |  |  |
| Current smoker, n (%) | 983 (48.7) | 52 (2.6) | 0.0356 | 1.58 (0.90, 2.76) | 0.1123 |
| Current drinker, n (%) | 1,430 (70.8) | 386 (19.1) | 0.0990 | 1.84 (1.40, 2.41) | <0.0001 |
| Physical inactivity, n (%) | 621 (30.7) | 640 (31.7) | 0.3972 | 6.09 (4.95, 7.50) | <0.0001 |
| Overweight/obesity, n (%) | 1,325 (65.6) | 1,133 (56.1) | 0.0941 | 1.49 (1.24, 1.79) | <0.0001 |
| Cardiometabolic disease |  |  |  |  |  |
| Hypertension, n (%) | 930 (46.0) | 826 (40.9) | 0.1974 | 2.25 (1.88, 2.70) | <0.0001 |
| Diabetes mellitus, n (%) | 457 (22.6) | 299 (14.8) | 0.1511 | 2.43 (1.87, 3.16) | <0.0001 |
| Dyslipidemia, n (%) | 878 (43.5) | 645 (31.9) | 0.0485 | 1.23 (1.02, 1.49) | 0.0292 |
| Hyperuricemia, n (%) | 454 (22.5) | 230 (11.4) | 0.0422 | 1.35 (0.99, 1.84) | 0.0584 |
|  |  |  |  |  |  |

Abbreviations: OR, odds ratio; CI, confidence interval.

**Table S2 |** Raw spearman correlations of cardiovascular risk factors within couples by age.

Qinghai, Gansu, Hebei, and Beijing, China, 2015-2019

| **Age (years)** | **No. of pairs** | **Risk factor** | | | | | | | | |
| --- | --- | --- | --- | --- | --- | --- | --- | --- | --- | --- |
|  |  | **BMI** | **SBP** | **DBP** | **FBG** | **TC** | **HDL-C** | **LDL-C** | **TG** | **UA** |
| Husbands |  |  |  |  |  |  |  |  |  |  |
| 20- | 616 | 0.08* | 0.11* | 0.09* | 0.25* | 0.19* | 0.13* | 0.08* | 0.16* | 0.22* |
| 50- | 1,404 | 0.21* | 0.23* | 0.14* | 0.23* | 0.15* | 0.06* | 0.18* | 0.18* | 0.16* |
| Wives |  |  |  |  |  |  |  |  |  |  |
| 20- | 678 | 0.09* | 0.12* | 0.09* | 0.26* | 0.18* | 0.14* | 0.08* | 0.20* | 0.22* |
| 50- | 1,342 | 0.21* | 0.22* | 0.14* | 0.21* | 0.15* | 0.05 | 0.17* | 0.16* | 0.16* |
| Abbreviations: BMI, body mass index; DBP, diastolic blood pressure; SBP, systolic blood pressure; DBP, diastolic blood pressure; FBG, fasting blood glucose; TC, total cholesterol; HDL-C, high-density lipoprotein cholesterol; LDL-C, low-density lipoprotein cholesterol; TG, triglycerides; UA, uric acid.  *: *P*<0.05 | | | | | | | | | | |

**Table S3 |** Spousal associations for different types of dyslipidemia by logistic regression analysis.

Qinghai, Gansu, Hebei, and Beijing, China, 2015-2019

| **Spouses’ status** | **Husbands** | |  | **Wives** | |  |
| --- | --- | --- | --- | --- | --- | --- |
|  | **Model 1** | **Model 2** |  | **Model 1** | **Model 2** |  |
| High TC |  |  |  |  |  |  |
| No (ref) | 1 | 1 |  | 1 | 1 |  |
| Yes | 1.73 (1.10, 2.71) | 1.40 (0.89, 2.22) |  | 1.74 (1.11, 2.74) | 1.47 (0.92, 2.33) |  |
| Low HDL-C |  |  |  |  |  |  |
| No (ref) | 1 | 1 |  | 1 | 1 |  |
| Yes | 1.47 (1.11, 1.95) | 1.39 (1.04, 1.86) |  | 1.47 (1.11, 1.95) | 1.37 (1.03, 1.83) |  |
| High LDL-C |  |  |  |  |  |  |
| No (ref) | 1 | 1 |  | 1 | 1 |  |
| Yes | 2.04 (1.14, 3.66) | 1.76 (0.98, 3.17) |  | 2.04 (1.14, 3.66) | 1.84 (1.02, 3.31) |  |
| High TG |  |  |  |  |  |  |
| No (ref) | 1 | 1 |  | 1 | 1 |  |
| Yes | 1.78 (1.39, 2.27) | 1.68 (1.30, 2.16) |  | 1.83 (1.43, 2.34) | 1.64 (1.27, 2.12) |  |
| Abbreviations: TC, total cholesterol; HDL-C, high-density lipoprotein cholesterol; LDL-C, low-density lipoprotein cholesterol; TG, triglycerides; ref, reference.  Values are presented as odds ratio (95% confidence interval) for having identical diseases among spouses.  Model 1 adjusted for age.  Model 2 further adjusted for education, annual income, geographic regions, smoking, drinking, leisure-time physical activity, and overweight/obesity. | | | | | | |

**Table S4 |** Sensitivity analysis on spousal associations for cardiovascular risk factors. Qinghai, Gansu, Hebei, and Beijing, China, 2015-2019

| **Spouses’ status** | **Husbands** | **Wives** |
| --- | --- | --- |
| Modifiable lifestyles ^a^ |  |  |
| Current smoker |  |  |
| No (ref) | 1 | 1 |
| Yes | 2.33 (1.22, 4.45) | 2.30 (1.20, 4.41) |
| Current drinker |  |  |
| No (ref) | 1 | 1 |
| Yes | 1.55 (1.15, 2.09) | 1.52 (1.13, 2.05) |
| Physical inactivity |  |  |
| No (ref) | 1 | 1 |
| Yes | 3.65 (2.84, 4.69) | 3.45 (2.69, 4.44) |
| Overweight/obesity |  |  |
| No (ref) | 1 | 1 |
| Yes | 1.36 (1.10, 1.68) | 1.38 (1.12, 1.72) |
| Cardiometabolic disease ^b^ |  |  |
| Hypertension |  |  |
| No (ref) | 1 | 1 |
| Yes | 1.13 (0.89, 1.43) | 1.04 (0.82, 1.33) |
| Diabetes |  |  |
| No (ref) | 1 | 1 |
| Yes | 1.74 (1.27, 2.37) | 1.54 (1.13, 2.09) |
| Dyslipidemia |  |  |
| No (ref) | 1 | 1 |
| Yes | 1.29 (1.04, 1.61) | 1.28 (1.04, 1.59) |
| Hyperuricemia |  |  |
| No (ref) | 1 | 1 |
| Yes | 1.36 (0.95, 1.95) | 1.34 (0.94, 1.91) |
| Abbreviations: ref, reference.  Values are presented as odds ratio (95% confidence interval) for having identical risk factors among spouses.  ^a^ Model adjusted for age, education, annual income, and geographic regions.  ^b^ Model adjusted for age, education, annual income, geographic regions, smoking, drinking, leisure-time physical activity, and overweight/obesity. Family history of hypertension and diabetes was additionally adjusted in the models of hypertension and diabetes, respectively. | | |
